# Application of a rank-based genetic association test to age-at-onset data from the Collaborative Study on the Genetics of Alcoholism study

**DOI:** 10.1186/1471-2156-6-S1-S53

**Published:** 2005-12-30

**Authors:** Yi-Ju Li, Eden R Martin, Ling Zhang, Andrew S Allen

**Affiliations:** 1 Center for Human Genetics, Duke University Medical Center, Durham, NC 27710, USA; 2 Department of Biostatistics, and Bioinformatics Duke University Medical Center, Durham, NC 27710, USA; 3 Duke Clinical Research Institute, Duke University Medical Center, Durham, NC 27710, USA

## Abstract

Association studies of quantitative traits have often relied on methods in which a normal distribution of the trait is assumed. However, quantitative phenotypes from complex human diseases are often censored, highly skewed, or contaminated with outlying values. We recently developed a rank-based association method that takes into account censoring and makes no distributional assumptions about the trait. In this study, we applied our new method to age-at-onset data on ALDX1 and ALDX2. Both traits are highly skewed (skewness > 1.9) and often censored. We performed a whole genome association study of age at onset of the ALDX1 trait using Illumina single-nucleotide polymorphisms. Only slightly more than 5% of markers were significant. However, we identified two regions on chromosomes 14 and 15, which each have at least four significant markers clustering together. These two regions may harbor genes that regulate age at onset of ALDX1 and ALDX2. Future fine mapping of these two regions with densely spaced markers is warranted.

## Background

Many statistical methods have been developed for linkage and association studies for both qualitative and quantitative traits [1-6]. Although quantitative traits are now recognized as important alternative phenotypes for gene mapping, association methods applications for qualitative traits are generally better developed than those for quantitative traits. One reason is that not all human complex diseases have appropriate quantitative measurements (phenotypes) that can be treated as genetic traits. Furthermore, many existing methods for quantitative traits assume normality of the data, which may not be appropriate when analyzing real data. For example, the distribution of a quantitative trait may be highly skewed, or right- or left-censored, making distribution-based methods inappropriate.

Age at onset is an important quantitative genetic trait for Alzheimer and Parkinson diseases [7]. Because age-at-onset data are measured in affecteds only, samples with phenotypic data are limited, thus reducing the power of association methods for quantitative traits. It would be desirable to incorporate information from unaffected siblings, because they may carry the risk genes but may not have reached disease onset. The age at onset of these unaffected individuals are censored.

We recently developed a new nonparametric association method that takes into account the censoring time of unaffected individuals [8]. We have conducted a series of simulation studies to evaluate the type I error and power of this new method. Our new method showed comparable statistical power with the method proposed by Monks and Kaplan [5] when quantitative traits without censoring were used. Substantial gains in power were found in our new method when censored individuals were included. The goal of this Genetic Analysis Workshop 14 (GAW14) genetic data analysis is to illustrate our new method on the age-at-onset data from the Collaborative Study on the Genetics of Alcoholism (COGA) dataset. We evaluate two age-at-onset traits: age at onset for ALDX1 and ALDX2. The age at interview variable was treated as the censoring time for unaffected individuals. We performed a genome-wide association analysis for age-at-onset traits using single-nucleotide polymorphisms (SNPs) from Illumina.

## Methods

### Rank-based association test

In order to reduce sensitivity to distributional assumptions and to include censored individuals, we developed a rank-based association test. This test can be applied to both case-parent (triad) data and sibships with or without parental genotypic information. Here, we describe the details of this new method.

We begin with one of the simplest pedigree structures: one offspring and two parents (triad). Let *T*_*i *_be the observation time (age-at-onset, age at exam, or age at death) of offspring *i*. Let *δ*_*i *_be a censored data indicator so that *δ*_*i *_= 1 when age at onset is observed and *δ*_*i *_= 0 when age at onset is censored (*T*_*i *_would then be the age at exam or age at death). Let *X*_*i *_be a coded vector for the genotype of offspring at a locus in the *i*^th ^family. Marker genotypes for the biallelic case are coded as described in Schaid [9] under different genetic models (general, dominant, recessive, and additive), in which the general model is a two degrees of freedom test using two indicator variables to express the marker genotypes. For the *i*^th ^family, form a vector of excessive transmission scores (*Z*_*i*_) by taking the coded offspring genotype and subtracting an average of possible coded genotypes given the parental data,



where  denotes the set of all coded offspring genotypes consistent with the genotypes of the parents. The *Z*_*i *_variable defined in this paper is analogous to the allelic transmission scores used by Monks and Kaplan [5] and Abecasis et al. [6].

Let *T*_(*l*) _represent the *l*^th ^of *k *ordered event times, *Z*_(*l*) _the excess transmission scores associated with *T*_(*l*)_, *m*_*l*_ the number of censored events in [*T*_*(l)*_, *T*_*(l+1)*_), and *n*_*l  *_the number of individuals at risk prior to *T*_(*l*)_, i.e., . The *i*^th ^triads score contribution takes into account the number of individuals at risk at each time point prior to the offspring's event time. Specifically,



For the case of multiple siblings with parental genotypes, we form a valid test by simply combining individual score contributions within a family. That is, we compute the score *U*_*ij *_for the *j*^th ^offspring in the *i*^th ^family as Equation 1, in which the rank of each event time is obtained by ordering the event times of all samples in the dataset. For sibship data without parental genotypes, the genotypic score (*Z*) is counted as the number of allelic differences among offspring. That is,



Again, the rank of each time event is based on all samples in the data set. Computing *U*_*ij *_for the *j*^th ^offspring in the *i*^th ^family is analogous to that described above with *Z*_*ij *_replacing *Z*_*i *_in Equation 1. The total score *U*_*i *_of family *i *is the sum of *U*_*ij *_across all *j *offsprings in family *i*.

Let *n *be the total number of families in the data set and . The variance of *U *can be estimated by the empirical variance



A score test for trait (age at onset) and genotype association can then be computed by

*W *= *U*'*V*^-^*U*,   (2)

where *V*^- ^denotes the generalized inverse of *V*. Asymptotically, *W*, is distributed as , where *p *is the rank of *V*.

### Analysis of COGA data

Age at onset for ALDX1 and ALDX2 and age at interview information from the COGA dataset were used as phenotypic data. ALDX1 was defined as an affected by the definitions of the DSM-III-R alcohol dependence and Feighner. ALDX2 was defined as an affected with DSM-IV alcohol dependence. For individuals without age-at-onset data for the trait, the individual was coded as censored and age at interview was treated as an event time. We developed a SAS program that implements the method described above and accommodates the pedigree structures of COGA data set.

We applied this method to the SNP dataset from Illumina linkage panel. We first analyzed whole-genome SNP data for the ALDX1 trait. Then, the chromosomes showing interesting results were followed up for the ALDX2 trait. The current SAS code is only suitable for the COGA pedigrees. A user-friendly program is still under development.

## Results

Our simulation studies demonstrated that the rank-based association test described above has correct type I error and higher statistical power than the Monks-Kaplan method [5] when censored rates are greater than 0 (manuscript in preparation). In this study, the traits of interest are the age at onset of ALDX1 and ALDX2 from COGA. The distributions of age at onset for ALDX1 and ALDX2 are similar and do not follow normal distribution. The skewness (kurtosis) was calculated as 1.96 (4.54) for age at onset of ALDX1 and 2.05 (5.37) for ALDX2. The average age at onset was 22.6 ± 8.3 for ALDX1 and 23.5 ± 8.5 for ALDX2. Because our proposed method does not assume normality for the trait distribution, it is still valid to apply our method to the raw data without transformation.

**Figure 1:**
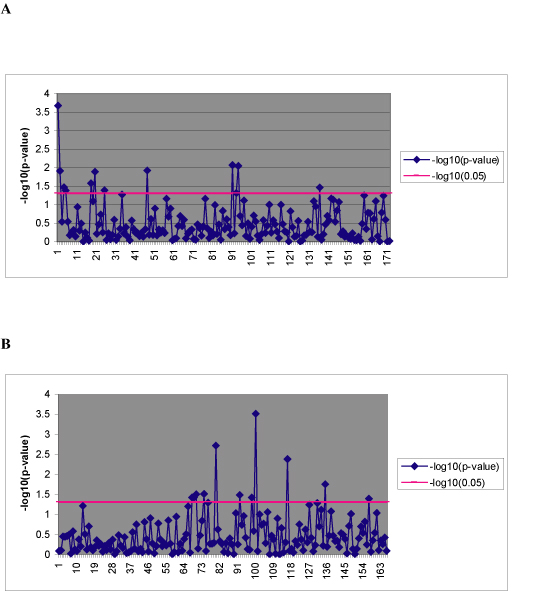
**Association results of all markers in chromosome 14 (A) and chromosome 15 (B). The *p*-values are in the form of -log_10_(*p*-value)**. The solid line indicates the cut-off for 0.05 significance level.

In total, 4,091 Illumina SNPs were analyzed for the age at onset of ALDX1. SNPs showing significant association with age at onset were scattered across all chromosomes (Table 1). On most chromosomes less than 5% of markers had significant *p*-values. Considering the significance level was set at 5%, we should interpret these results carefully. Seven chromosomes (chromosome 8, 9, 10, 13, 14, 15, and 21) had more than 5% of markers significant. Two SNPs, on chromosome 14 and 15, respectively, showed strong association with age at onset (*p *= 0.0002 and 0.0003). In addition, we observed a pattern of at least four significant SNPs clustering together on both chromosomes 14 and 15, which is depicted in Figure 1. The interesting chromosomal regions were from 0 cM (rs1972373) to 0.6 cM (rs1760912) on chromosome 14 and 47.6 cM (rs1864299) to 61 cM (rs749468) on chromosome 15. The same significant markers on chromosome 14 and 15 were identified when age-at-onset data of ALDX2 were analyzed. Overall, these results suggest some potential areas of interest on these two chromosomes.

## Discussion

Our goal for this GAW workshop was to illustrate a new association method that we recently developed for age-at-onset traits in a real data set. Through this project, we developed a SAS program to analyze the COGA data. This exercise will help us toward developing a user-friendly program.

In this study, we focused on age at onset of the ALDX1 and ALDX2 traits. Because our new method can be applied to any quantitative trait regardless of underlying distribution, it is applicable to the highly skewed age-at-onset data observed in the COGA dataset. Our genome-wide association tests for age at onset of ALDX1 using Illumina SNPs showed a very low percentage of significant makers: only 206 of 4,091 markers reached the significance level of 0.05. Due to the large number of markers tested, multiple corrections should be taken into account. Therefore, the percentage of significant markers was reduced further. One possible explanation is that this SNP chip was not designed for association analysis, because the SNPs are not densely distributed. Many association studies test markers spaced between 20 and 50 kilobases apart in order to detect significant association. Therefore, we did not expect a high percentage of significant results.

Our analysis showed that both chromosomes 14 and 15 have more than 5% of the markers significantly associated with age at onset of both ALDX1 and ALDX2. In addition, on both chromosomes the marker with the strongest association signal clusters with other significant markers in a small region (0.6 cM for chromosome 14 and 13.4 cM for chromosome 15). These two potential candidate regions may harbor genes that regulate age at onset of ALDX1 and ALDX2. It will be worthwhile to follow up these two regions with dense markers in the future.

In our analysis of chromosomes 14 and 15, we did not find different association patterns for age at onset between ALDX1 and ALDX2. This is mainly due to the similar distribution of age at onset between these two phenotypes. Many individuals were recorded to have the same or similar onset time for ALDX1 and ALDX2. The maximum difference between these two phenotypes within the same individual was 8 years. This points out a challenge for obtaining accurate age-at-onset data in this study. Since ALDX1 and ALDX2 were defined by the severity of alcohol dependence, it is possible that the similar onset time for these two phenotypes reflects the fact that they are modified by same genetic mechanism. However, it is also possible that a participant cannot easily separate the onset time of these similar clinical features.

## Abbreviations

COGA: Collaborative Study on the Genetics of Alcoholism

GAW14: Genetic Analysis Workshop 14

SNP: Single-nucleotide polymorphism

## Figures and Tables

**Table 1:** Summary of association test for age at onset of ALDX1 using Illumina

	SNPs			
		
Chromosome	Total	No. significant^a^	% significant	Minimum *p*-value
1	250	9	3.6	0.009
2	250	13	5.2	0.009
3	250	10	4.0	0.002
4	250	12	4.8	0.001
5	250	12	4.8	0.003
6	250	8	3.2	0.003
7	250	11	4.4	0.002
8	211	13	**6.2**^b^	0.010
9	176	12	**6.8**	0.008
10	207	14	**6.8**	0.004
11	186	7	3.8	0.002
12	231	7	3.0	0.020
13	167	12	**7.2**	0.003
14	172	12	**7.0**	**0.0002**
15	166	11	**6.6**	**0.0003**
16	162	7	4.3	0.003
17	132	4	3.0	0.001
18	131	6	4.6	0.006
19	110	5	4.5	0.001
20	118	12	1.02	0.002
21	87	6	**6.9**	0.020
22	85	3	3.5	0.020

^a^*p *≤ 0.05

^b^Bold text indicates chromosomes for which either the percentage of significantly associated SNPs was greater than 6% or the minimum *p*-value was ≤ 0.0003.
